# Global Developments in Priority Setting in Health

**DOI:** 10.15171/ijhpm.2017.10

**Published:** 2017-01-28

**Authors:** Rob Baltussen, Craig Mitton, Marion Danis, Iestyn Williams, Marthe Gold

**Affiliations:** ^1^Radboud University Medical Center, Nijmegen, The Netherlands.; ^2^University of British Columbia, Vancouver, BC, Canada.; ^3^National Institutes of Health, Bethesda, MD, USA.; ^4^University of Birmingham, Birmingham, UK.; ^5^New York Academy of Medicine, New York City, NY, USA.; ^6^City College, New York City, NY, USA.

**Keywords:** Priority Setting, Legitimacy, Stakeholder Participation

## Abstract

Countries around the world are experiencing an ever-increasing need to make choices in investments in health and healthcare. This makes it incumbent upon them to have formal processes in place to optimize the legitimacy of eventual decisions. There is now growing experience among countries of the implementation of stakeholder participation, and a developing convergence of methods to support decision-makers within health authorities in making tough decisions when faced with the stark reality of limited resources. We call for further interaction among health authorities, and the research community to develop best practices in order to confront the difficult choices that need to be made.

Countries around the world experience an ever-increasing need to make choices in healthcare. In high-income countries, the rapid development of expensive new drugs demands a growing share of the available budgets. For example, in the United States, costs of the drug Sovaldi for the treatment of Hepatitis C are as high as US$84 000 per patient, and may amount to a total budget impact of US$65 billion in the next 5 years.^1^ Coupled with rising public expectations and fiscal austerity, this leads to a situation of fiercely competing demands on available budgets. Lower-income countries, having a higher burden of disease but lower available budgets, are even more challenged on their path towards universal health coverage.^2^

In this context, more than 200 delegates from 22 countries convened in September 2016 in Birmingham, UK, at the 11th International Conference on Priorities in Health. Academics from various disciplines including medicine, ethics, public health, health economics, political and management sciences, together with policy-makers and patients, discussed best practices for setting priorities to inform coverage decisions on health interventions. Discussions at the conference centred around four themes.

Firstly, as signalled in a recent World Health Organization (WHO) survey among 111 countries, the majority of countries has formal processes in place to collect and analyse information for making coverage decisions.^3^ Yet, many nations still lack well-defined processes for considering evidence in decision-making. Their coverage decisions are therefore at risk of being ad-hoc and difficult to justify. At the conference, a pervasive message for health authorities in countries around the world was to put formal processes in place.

Second, health authorities use different criteria in these formal processes. The WHO survey reported that in making coverage decisions on pharmaceuticals, most countries ‘always or almost always’ considered safety and clinical effectiveness. Yet, less than half of countries considered economic aspects, and only a few countries considered acceptability to healthcare providers and patients, equity issues, ethical issues and feasibility considerations as such.^3^ At the conference, it was emphasised that countries can learn from each others’ experiences on the use of criteria for coverage decisions, as well as from international recommendations.^2^

Third, countries make different choices on how they organise their processes, for example in terms of stakeholder involvement of patients and health professionals, transparency of the process, and whether or not to allow for appeals. If processes omit stakeholder involvement and/or lack transparency this can compromise the extent to which members of society accept health authorities as moral authorities, and whether they consider health authority decisions to be legitimate. The growing litigation in a country like Mexico, where citizens challenge negative reimbursement decisions on health interventions as taken by health authorities in court in order to acquire access to these interventions, may be seen as a manifestation of this.^4^ At the conference, meaningful stakeholder involvement was stressed as an important prerequisite for coverage decision-making, as was the need to design health authorities with the necessary resources and credibility to make defensible decisions.^5^ Again, countries can learn from each others’ processes, and the work of the International Decisions Support Initiative^6^ in lower-income countries and the European Network on Health Technology Assessment^7^ are both instrumental in this respect.

Fourth, there is a developing convergence of methods to support authorities in setting priorities. Cost-effectiveness analysis (CEA) has long been the principal method for doing so, and remains relevant. Yet, the use of CEA for priority setting is increasingly being cited for its narrow emphasis on efficiency, and as such, for insufficiently taking into account other relevant stakeholder values.^8^ At the same time, ethical frameworks for priority setting have been developed, centred around stakeholder deliberation.^9^ These twin issues are being addressed currently through a range of methods that can be classified under the general heading of ‘evidence-informed deliberative processes.’^8,10,11^ The methods, are, on the one hand, based on deliberation between stakeholders to identify, reflect and learn about the meaning and importance of their respective values. On the other hand, they are based on rational decision-making – this involves the interpretation of evidence that is available (or is being additionally collected), and which may further shape the deliberation on the values. An important principle here is evidence-informed evaluation, which allows contributions of stakeholders in terms of their personal) experiences and judgments.^8^ At the conference, delegates considered this developing convergence on methods and its broad implementation as highly encouraging. Yet, it should be realized that priority setting is not only about developments of methods. It is also about how to create the context where these are used – eg, the organisation, development and building of institutions and institutional support systems, and management of power relations.^12,13^

In summary, the pressing need for nations to allocate resources such that health is maximized in a fashion that is acceptable to its populations makes it incumbent upon countries to have formal processes in place to optimize the legitimacy of eventual decisions. There is now growing experience among countries of the implementation of such processes, and a developing convergence of methods to support decision-makers within health authorities in making tough decisions when faced with the stark reality of limited resources. We call for further interaction among health authorities and the research community to develop best practices in order to confront the difficult choices that need to be made.

## Acknowledgments

We thank Kjell Arne Johansson and Maarten Jansen for their comments on earlier version of this manuscript.

## Ethical issues

Not applicable.

## Competing interests

All authors have completed the ICMJE uniform disclosure form at http://www.icmje.org/coi_disclosure.pdf and declare: no support from any organisation for the submitted work; no financial relationships with any organisations that might have an interest in the submitted work in the previous three years no other relationships or activities that could appear to have influenced the submitted work.

## Authors’ contributions

All authors contributed equally to the writing of this paper.

## Authors’ affiliations

^1^Radboud University Medical Center, Nijmegen, The Netherlands. ^2^University of British Columbia, Vancouver, BC, Canada. ^3^National Institutes of Health, Bethesda, MD, USA. ^4^University of Birmingham, Birmingham, UK. ^5^New York Academy of Medicine, New York City, NY, USA. ^5^City College, New York City, NY, USA.
